# Arachidonic acid metabolism in human prostate cancer

**DOI:** 10.3892/ijo.2012.1588

**Published:** 2012-08-10

**Authors:** PEIYING YANG, CARRIE A. CARTWRIGHT, JIN LI, SIJIN WEN, INA N. PROKHOROVA, IMAD SHUREIQI, PATRICIA TRONCOSO, NORA M. NAVONE, ROBERT A. NEWMAN, JERI KIM

**Affiliations:** 1 Departments of General Oncology; 2 Cancer Biology; 3 Systems Biology; 4 Biostatistics; 5 Pathology; 6 Clinical Cancer Prevention; 7 Genitourinary Medical Oncology and; 8 Experimental Therapeutics, The University of Texas MD Anderson Cancer Center, Houston, TX, USA

**Keywords:** arachidonic acid, cyclooxygenase, lipoxygenase, prostate cancer, xenograft, eicosanoid

## Abstract

The arachidonic acid pathway is important in the development and progression of numerous malignant diseases, including prostate cancer. To more fully evaluate the role of individual cyclooxygenases (COXs), lipoxygenases (LOXs) and their metabolites in prostate cancer, we measured mRNA and protein levels of COXs and LOXs and their arachidonate metabolites in androgen-dependent (LNCaP) and androgen-independent (PC-3 and DU145) prostate cancer cell lines, bone metastasis-derived MDA PCa 2a and MDA PCa 2b cell lines and their corresponding xenograft models, as well as core biopsy specimens of primary prostate cancer and nonneoplastic prostate tissue taken *ex vivo* after prostatectomy. Relatively high levels of COX-2 mRNA and its product PGE_2_ were observed only in PC-3 cells and their xenografts. By contrast, levels of the exogenous 12-LOX product 12-HETE were consistently higher in MDA PCa 2b and PC-3 cells and their corresponding xenograft tissues than were those in LNCaP cells. More strikingly, the mean endogenous level of 12-HETE was significantly higher in the primary prostate cancers than in the nonneoplastic prostate tissue (0.094 vs. 0.010 ng/mg protein, respectively; p=0.019). Our results suggest that LOX metabolites such as 12-HETE are critical in prostate cancer progression and that the LOX pathway may be a target for treating and preventing prostate cancer.

## Introduction

Rates of prostate cancer incidence vary dramatically in different geographic locations (1,2). Dietary fat intake, which is sometimes used to help explain this variance, is among the most widely studied dietary risk factors for prostate cancer, yet its role in influencing cancer risk remains controversial (3). We do know that dietary fat composition may greatly influence the risk of prostate cancer (4) as it has been observed in prostate cancer cell lines that n-6 fatty acids, such as linoleic acid and arachidonic acid (AA), promote cell proliferation, whereas long-chain polyunsaturated n-3 fatty acids, which are abundant, for example, in fish oil, inhibit cell proliferation (5–8). These promotional and inhibitory effects of n-6 and n-3 fatty acids, respectively, have also been demonstrated in prostate carcinogenesis and progression *in vivo* (9). Evidence is also available from specimens obtained during radical prostatectomy that AA turnover is 10 times greater in tumor tissue than it is in normal prostate tissue (7).

Of the possible mechanisms responsible for AA-induced cell proliferation in prostate cancer, one of the most likely is the generation of certain eicosanoids, key mediators of the inflammatory response (10). They are produced by both tissue cells and tumor-infiltrating leukocytes (10), and they may act as autocrine and/or paracrine factors. Synthesized from polyunsaturated fatty acids, eicosanoids play important biologic roles in cell proliferation and tissue repair, blood clotting, blood vessel permeability, inflammation, and immune cell behavior (10). They fall into three general groups: prostaglandins (PGs), including PGE_2_; leukotrienes (LTs); and thromboxanes. They also are associated with the enzymatic pathways of the cyclooxygenases (COX-1 and COX-2), lipoxygenases (LOXs), and cytochrome P450.

The PGs are synthesized by the action of COX enzymes: the constitutively expressed COX-1 and the inducible COX-2. COX-2 can be stimulated by inflammatory mediators, cytokines, growth factors, and tumor promoters and can be inhibited by steroids and certain nonsteroidal anti-inflammatory drugs (11). Higher than usual COX-2 and PGE_2_ levels have been observed in numerous malignancies, including those of the colon, lung, head and neck, breast, pancreas, bladder, and prostate (11). Both AA and PGE_2_ stimulate cell proliferation and tumor growth *in vitro* in PC-3 human prostate cancer cells (12). In PC-3, LNCaP, and DU145 prostate cancer cell lines, upregulation of COX-2 and PGE_2_ has been inversely correlated with apoptosis (13).

Relative to the effects of the PGs, those of the LOX products, such as LTs and hydroxyeicosatetraenoic acids (HETEs), are more diverse and may be cell-type specific. An AA metabolite derived from 12-LOX, 12-HETE, promotes the proliferation of human colon, pancreatic, and breast cancer cell lines and plays an important role in cell adhesion and promotion of metastasis (14). Expression of 12-LOX also appears to stimulate angiogenesis in human prostate carcinoma cells (14). More recently, study results have suggested that 12-LOX functions as a potential biomarker and therapeutic target for prostate cancer stem cells (15). Further, the 5-LOX product 5-HETE has been suggested as playing a role as a potent pro-growth survival factor for human prostate cancer cells (16).

By contrast, we and others have reported that 15-LOX-1, 15-LOX-2, and their related products 13-hydroxyoctadecadienoic acid (13-HODE) and 15-HETE actually function as tumor suppressors in, respectively, colorectal and prostate cancer cells (17,18). In addition, 15-deoxy-Δ^12,14^-PGJ_2_, the metabolite of PGJ_2_ and a peroxisome proliferator-activated receptor γ (PPARγ) ligand, has been reported to induce cell death in three human prostate cancer cell lines, PC-3, LNCaP, and DU145 (19).

Thromboxanes, additional metabolites of COX enzymes, have also been studied in cancer. For example, higher thromboxane synthase mRNA levels have been observed in prostate and uterine cancers relative to levels in matched normal tissues (20). In prostate cancer, higher expression of thromboxane synthase was associated with advanced-stage and high-grade disease (21). Thromboxane synthase expressed in prostate cancer cells was enzymatically active and might play a contributory role in tumor progression, especially in tumor cell motility (22,23).

*In vitro* studies have demonstrated that eicosanoids appear to have key roles in the biology of human prostate cancer, but the role of endogenous eicosanoids *in vivo* in prostate cancer development and progression remains unclear. We and others have investigated the alteration of AA metabolites in the TRAMP mouse model and found that 12-HETE and COX-2 levels were significantly higher in prostate tissues of these mice than they were in those of control or wild-type mice (24,25). However, endogenous PGE_2_ was almost five times lower in the prostate of TRAMP mice than it was in wild-type mice (24).

Shappell *et al* (26) found in human samples from radical prostatectomy specimens that expression of COX-2 was elevated only in high-grade tumors. By comparison, the reduction of 15-LOX-2 and 15-HETE formation is the most characteristic alteration of AA metabolism in prostate cancer (26). We also reported that 15-LOX-2 expression was lost in all the prostate cancer cell lines we tested (including PC-3, LNCaP, and DU145 cells), yet easily detectable levels of 15-LOX-2 were expressed in normal human prostate cells (17,27). Not surprisingly, eicosanoid profiles differ between cancer and normal prostate cells and between mouse and human prostate tissues.

Changes in eicosanoid content appear to be important for a more complete understanding of prostate cancer progression. We therefore undertook this study in androgen-dependent and androgen-independent cell lines from bone, brain, and lymph node metastases; in rodent xenografts of human tumors with bone microenvironment influence; and in human tumor tissues. Our findings from these studies show that eicosanoids may play an important role in prostate cancer progression in a cell type- and microenvironment-dependent manner.

## Materials and methods

### Eicosanoids and antibodies

The eicosanoids PGE_2_, 5-HETE, 12-HETE, and 15-HETE, and the corresponding deuterated eicosanoid standards were purchased from Cayman Chemical. AA, butylated hydroxytoluene (BHT), citric acid, and EDTA were obtained from Sigma Chemical. All high-pressure liquid chromatography (HPLC) solvents used for analyses of eicosanoids were purchased from Fisher Scientific. Anti-COX-2 and anti-15-LOX antibodies were obtained from Cayman Chemical. Anti-5-LOX antibody was purchased from Research Diagnostics and anti-β-actin antibody was purchased from Sigma Chemical. Anti-platelet-type 12-LOX antibody was obtained from Oxford Biomedical Research.

### Cell lines

Human prostate cancer cell lines LNCaP, PC-3, and DU145 were obtained from the American Type Culture Collection and maintained in a humidified atmosphere containing 5% CO_2_ at 37°C. LNCaP and PC3 cells were routinely cultured in RPMI-1640 medium (Invitrogen), supplemented with 10% heat-inactivated fetal bovine serum (FBS) (Hyclone Laboratories) containing 50 IU/ml penicillin, 50 μg/ml streptomycin, and 2 mM L-glutamine [all from Gibco (Invitrogen)]. DU145 cells were cultured under similar conditions except that cells were grown in DMEM medium (Mediatech).

The human prostate cancer cell lines MDA PCa 2a and MDA PCa 2b were gifts from Dr Nora Navone (28). These cell lines were grown in BRFF-HPC1 medium (AthenaES) supplemented with 20% FBS in tissue culture flasks coated with FNC coating mix (AthenaES). All cell lines were authenticated via microscopic morphology check and DNA analysis.

### Human prostate xenografts

All animal experiments were approved by the Institutional Animal Care and Use Committee at MD Anderson. Xenograft models of PC-3, LNCaP, DU145, and MDA PCa 2b cells were developed as previously described (28). In brief, PC-3 and DU145 (1×10^6^), LNCaP (5×10^6^), and MDA PCa 2b (1×10^7^) cells were suspended in 50% Matrigel (BD Biosciences) and subcutaneously implanted into the flanks of 8-week-old male BALB/c athymic (Nu/Nu) mice. Mice were euthanized with CO_2_ approximately 1 month later, when the tumor volume reached 1 cm^3^. A portion of each tumor was snap frozen and stored at −80°C until measurements of eicosanoid profiles and protein expression of eicosanoid enzymes were obtained.

### Human core biopsy specimens from prostatectomy specimens

*Ex vivo* biopsy specimens were obtained from eight patients undergoing prostatectomy who consented to the use of their tissues in research, according to the Institutional Tissue Banking protocol at MD Anderson. Whenever possible, multiple core biopsy specimens were obtained from each prostatectomy specimen. The presence of tumor in each core was determined by making a touch prep of the cores before the specimens were snap frozen in liquid nitrogen, placed in cryovials, and archived at −80°C in the Prostate Tissue Bank.

Technicians ensured conformance with the prostatectomy specimen map delineating individual tumor foci. Seven core biopsy specimens containing tumor and three without tumor were used to test for the expression of COXs and LOXs; seven core biopsy specimens without tumor and 19 with tumor were used for evaluating eicosanoid profiles. In this proof-of-principle analysis, the limited amount of tissue prevented the testing of matching specimens across all two-sample types and evaluating intrapatient as well as interpatient variabilities.

### RNA extraction and real-time polymerase chain reaction

Total RNA was extracted from the five prostate cancer cell lines using TRI reagent (Molecular Research Center). The RNA from each sample was reverse transcribed and then measured quantitatively using real-time polymerase chain reaction (PCR) and a comparative threshold cycle method, as previously described (29,30).

### Western blot analysis

Cells growing in log phase were washed with cold PBS and scraped free in the presence of a lysis buffer (Invitrogen) with protease inhibitor cocktail (Sigma). Cell lysates were then sonicated on ice for 3 min, incubated for 10 min on ice, and centrifuged at 14,000 × g for 10 min at 4°C.

Protein levels were quantified via the detergent-compatible protein assay (Bio-Rad). Equal levels of protein (60 μg) were fractionated on precast gels (Bio-Rad) and then transferred onto polyvinylidene diflouride membranes, according to standard methods. After a 1- to 2-h incubation in 5% nonfat dry milk blocking buffer prepared in Tris-buffered saline with 0.1% Tween 20, the membranes were probed with primary antibodies diluted 1:2,000 in blocking buffer. Protein bands were visualized via chemiluminescence, using the ECL+ detection kit (Amersham Biosciences) and hyper-film (Amersham Biosciences). Equal loading of samples was illustrated by Western blotting for the presence of β-actin.

### Eicosanoid analyses

The levels of eicosanoids in the prostate cancer cells, xenograft tissues, and human biopsy samples were determined according to the methods of Kempen *et al* and Yang *et al* (31–33). In brief, the human prostate cancer cells (5×10^6^) were harvested by trypsinization, washed with PBS, and then resuspended in 0.5 ml of PBS containing 1 mM CaCl_2_. For exogenous eicosanoid analysis, samples were incubated with 2.5 μl of calcium ionophore A23187 (1 mM; Sigma) for 2 min at 37°C, followed by addition of an aliquot of 2.5 μl of 10 mM AA. Samples were then incubated for a further 10 min under similar conditions. The reaction was terminated by the addition of aliquots of 40 μl of 1 N citric acid and 5 μl of 10% BHT. An aliquot of 10 μl of the relevant deuterated eicosanoids (PGE_2_-d_4_, 15-HETE-d_8_, 12-HETE-d_8_, and 5-HETE-d_8_) per 100 ng/ml) was added to the reaction mixtures as internal standards. The eicosanoids were extracted three times with 2 ml of hexane-ethyl acetate (1:1, v/v). The upper organic phases were then pooled and evaporated to dryness under a stream of nitrogen at room temperature. All extraction procedures were performed under conditions of minimal light. Samples were then reconstituted in 100 μl of methanol with 10 mM ammonium acetate buffer (70:30, v/v; pH, 8.5) before analysis by liquid chromatography/tandem mass spectrometry (LC/MS/MS). For the measurement of endogenous eicosanoids, the resuspended cells were acidified by the addition of an aliquot of 40 μl of 1 N citric acid followed by the addition of the deuterated internal standards and extraction of eicosanoids as previously described (33).

For analysis of eicosanoids in the xenograft tissues, each frozen tissue specimen (25–50 mg) was ground to a fine powder in a liquid nitrogen-cooled mortar (Fisher). Samples were then transferred to microcentrifuge tubes. Ice-cold PBS buffer, triple each sample’s volume and containing 0.1% BHT and 1 mM EDTA, was added, and the tubes were sealed. The mixtures were then homogenized using an ultrasonic processor (Misonix) at 0°C for 3 min. A 100-μl aliquot of each homogenate was transferred to a glass tube (13×100 mm), and the eicosanoids were extracted using the method of Yang *et al* (33).

We next measured the AA metabolites in the human prostate core biopsy samples using a modification of the method of Yang *et al* (33). In brief, the frozen specimens were mixed with 150 μl of homogenization buffer [500 mM Tris-HCl (pH 7.2), 0.5 M sucrose, 200 M EDTA, 100 mM EGTA, 0.4 M NaF, 10% Triton X-100, and 10 mM sodium orthovanadate (Sigma)] and incubated at 0°C for 30 min. The samples were then homogenized and processed for eicosanoid extraction as described above.

Reverse-phase HPLC electrospray ionization MS was used to measure eicosanoid (PGE_2_, 5-HETE, 12-HETE) levels in cells using our previously published method (31,34). A Micromass Quattro Ultima tandem mass spectrometer (Waters) was equipped with an Agilent 1100-HP binary pump HPLC inlet for use in these studies. Eicosanoids were separated by a Luna 3-μ phenyl-hexyl (2×150 mm) LC column (Phenomenex). The mobile phase consisted of 10 mM ammonium acetate (pH 8.5) and methanol; the flow rate was 250 μl/min, the column temperature was 50°C, and the sample injection volume was 25 μl. Samples were kept at 4°C in an autosampler before their injection into the analytical column. The mass spectrometer was operated in the electrospray negative-ion mode with a cone voltage of 100 V. Fragmentation of all compounds was performed using argon as the inert collision gas at a cell pressure of 2.1×10^−3^ torr. The collision energy was 19 V. All eicosanoids were detected using negative ionization and multiple-reaction monitoring of the transition ions for eicosanoid products and their internal standards (33).

### Statistical analyses

Descriptive statistics were calculated and exploratory data analyses were performed. Categorical data were summarized by frequency counts, proportions, or percentages. Continuously scaled measures were summarized as means and standard deviations or as medians with ranges. Gene expression image plots, bar graphs, and scatter plots were used to graphically present the data. Student’s t-test was used for analyzing continuous variables. A mixed-effects model was used in the analysis of repeated measurements from the same patients. All statistical tests were two sided, and p-values of <0.05 were considered to indicate statistically significant differences. Statistical analyses were performed with S-PLUS software (TIBCO Software).

## Results

### AA metabolism enzyme expression in human prostate cancer cell lines

**Figure 1:**
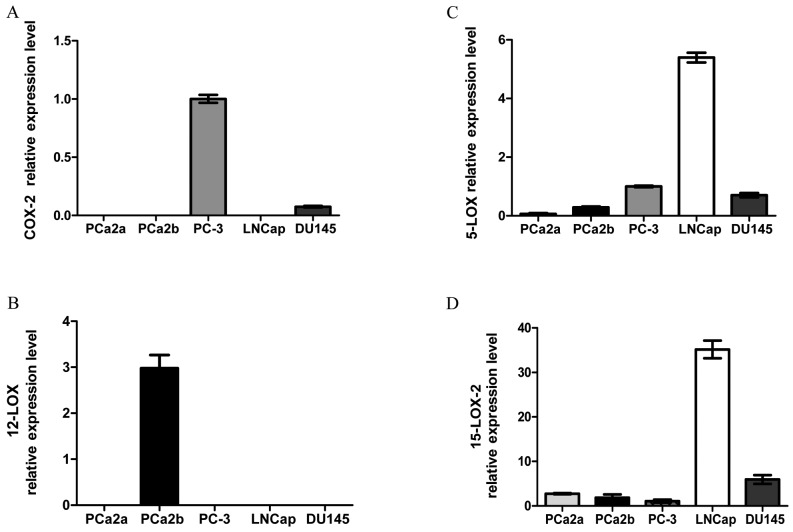
Quantitative analysis of mRNA of eicosanoid enzymes in five human prostate cancer cell lines. Cells (1×10^6^) were plated in 100-mm dishes and allowed to attach overnight. They were then collected and subjected to RNA extraction as described in Materials and methods. The expression levels were calculated as the values relative to that of a calibrator sample (PC-3). Data are presented as the means ± SDs of three separate experiments.

The data from the quantitative analysis of mRNA of eicosanoid enzymes in the five different human prostate cancer cell lines are shown in Fig. 1. COX-2 mRNA was detectable only in PC-3 and DU145 cells, with PC-3 cells containing 10 times more than DU145 cells (Fig. 1A). By contrast, levels of 12-LOX mRNA were observed only in the MDA PCa 2b metastatic prostate cancer cells (Fig. 1B). Notably, the level of 5-LOX mRNA in LNCaP cells was almost five times higher than that in PC-3 cells, whereas very limited expression of 5-LOX was detected in MDA PCa 2a and MDA PCa 2b cells (Fig. 1C). The mRNA level of 15-LOX-2 was also highest in the LNCaP cells, relative to that in the other cells tested (Fig. 1D).

### Expression of COX-2 and the LOXs in prostate cancer cell lines

**Figure 2:**
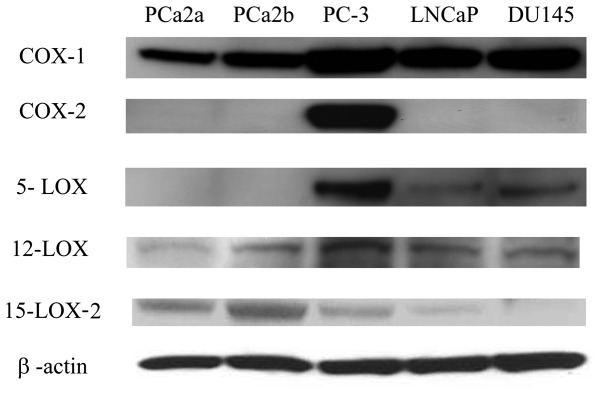
Protein expression of the eicosanoid enzymes in prostate cancer cells. Cells that reached approximately 80% confluency were collected and lysed in the lysis buffer as described in Materials and methods. The expression of protein was determined by Western blot analysis using their relevant antibodies. Data are presented as representative blots of replicate experiments.

The protein expression of COX-2 and LOX enzymes was examined in the five different prostate cancer cell lines by Western blotting to confirm that the RNA expression of these enzymes had resulted in protein expression. Although the protein expression of COX-2 in PC-3 cells was similar to that of mRNA in this cell line, the protein expression levels of 5-LOX, 12-LOX, and 15-LOX-2 were somewhat different from that of mRNA (Fig. 2). This could be reflective of either assay differences or, more importantly, differences in mRNA translation efficiency between the various cancer cell lines.

### Endogenous and exogenous AA metabolism in the human prostate cancer cell lines

**Figure 3:**
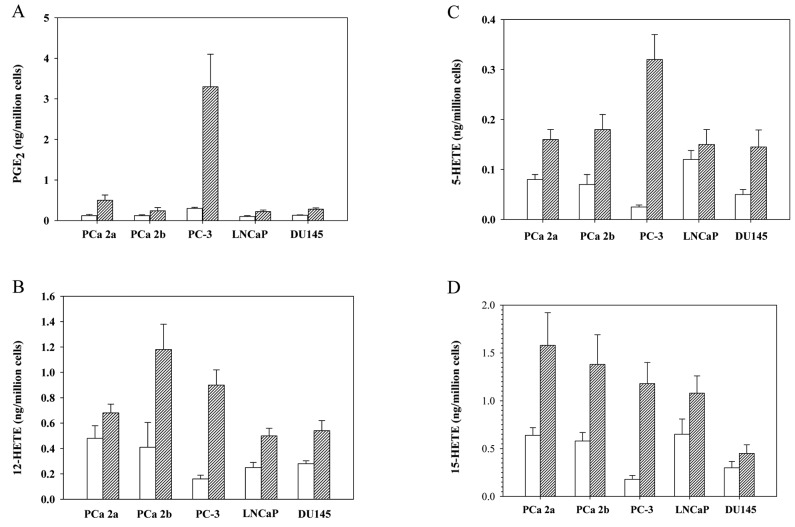
Endogenous (clear bars) and exogenous (striped bars) eicosanoid metabolism in the five human prostate cancer cell lines. For the endogenous eicosanoid analysis, cells (3×10^6^) were plated and allowed to attach overnight. They were then harvested by trypsinization and subjected to eicosanoid analysis using LC/MS/MS, as described in Materials and methods. For the exogenous eicosanoid analysis, cells (5×10^6^) were treated with 50 μM arachidonic acid for 10 min and then analyzed for eicosanoid content. Data are presented as the means ± SDs of three separate experiments.

The mRNA and protein levels of each eicosanoid enzyme were differently regulated in the five prostate cancer cell lines we tested, which suggested that the relevant metabolites of the eicosanoids may also differ. Therefore, we measured both endogenous and exogenous eicosanoid levels in those cell lines. As shown in Fig. 3A, levels of both endogenous and exogenous PGE_2_, a COX-2 metabolite, were highest in the PC-3 cells, suggesting a high capacity to produce this pro-inflammatory eicosanoid. The level of exogenous PGE_2_ in PC-3 cells was almost 15 times higher than that produced endogenously.

By comparison, the levels of endogenous 12-HETE in the metastatic prostate cancer cells MDA PCa 2a (0.48±0.10 ng/million cells) and MDA PCa 2b (0.41±0.19 ng/million cells) were almost twice those in the PC-3, DU145, and LNCaP cells (0.16–0.25 ng/million cells) (Fig. 3B). When AA was used as a supplement for the cells, the formation of 12-HETE was markedly increased in the MDA PCa 2b and PC-3 cells. The level of exogenous 12-HETE was ranked from highest to lowest in the MDA PCa 2b, PC-3, MDA PCa 2a, DU145, and LNCaP cells.

The level of endogenous 5-HETE was highest in the LNCaP cells, but the exogenous level of 5-HETE was noticeably higher in the PC-3 cells, in fact, more than 1.5 times the levels in the other cell lines (Fig. 3C). This suggests that the capacity to produce 5-LOX is greater in PC-3 cells than in the other cancer cell lines tested, and it correlated with the highest expression of the 5-LOX protein in this particular cell line (Fig. 2). The level of endogenous 15-HETE was also highest in LNCaP cells, but after exposure of the cell lines to AA, the MDA PCa 2a cells showed the highest level of 15-HETE formation (Fig. 3D).

### Expression of COX-2 and the LOXs in human prostate cancer xenograft tissues

**Figure 4:**
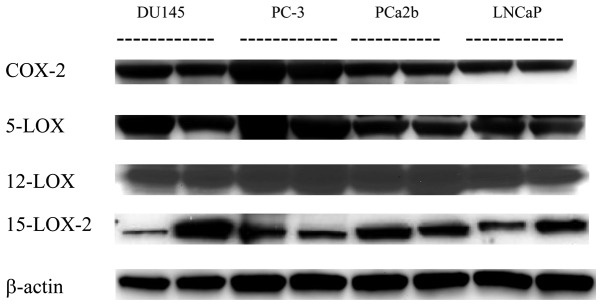

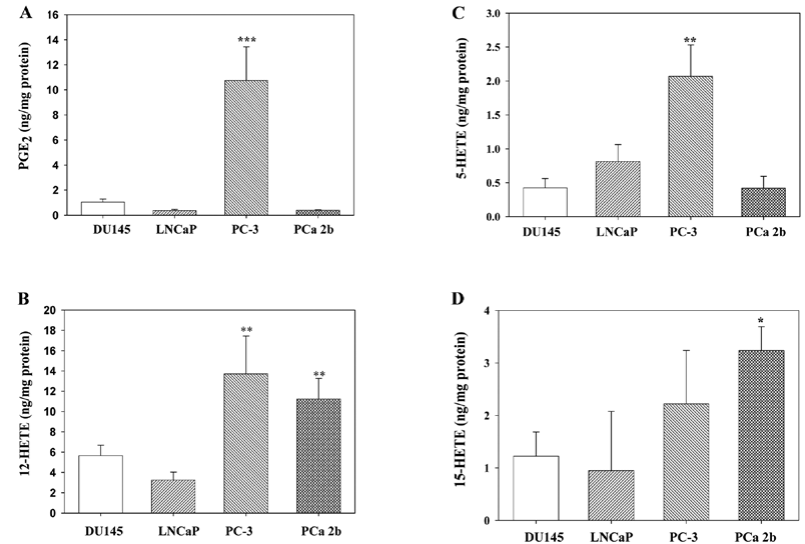
Expression of the eicosanoid enzymes (top panel) and their relevant products in human prostate mouse xenograft tissues (bottom panel). Tumor tissues were collected when the tumor volume was approximately 1 cm^3^. Protein levels were detected by Western blotting with relevant antibodies (top panel). Flash-frozen tissues were pulverized and extracted for the endogenous eicosanoid levels by LC/MS/MS. The levels of eicosanoids were normalized by protein concentrations (bottom panel, A–D). Data are presented as the means ± SDs in each group (n≥5). ^*^p<0.05, ^**^p<0.01 vs. LNCaP xenograft tissues. ^***^p<0.001 vs LNCaP, DU145 and PCa 2b xenograft tissues.

To explore the role of eicosanoids in prostate cancer progression, we further investigated the expression of COX-2 and various LOX proteins in human prostate xenograft tissues (Fig. 4, top panel). As the tumorigenicity of the MDA PCa 2a cells was extremely low, we evaluated the protein expression of eicosanoid enzymes in only the other four human prostate cancer xenograft tissues. The protein expression of the COX-2 and 5-LOX enzymes was highest in the PC-3 xenografts, whereas the protein levels of 12-LOX were higher in both PC-3 and PCa 2b cells. In line with the protein expression of 15-LOX-2 in the cells *in vitro*, its expression was consistently highest in the two tumor tissues derived from MDA PCa 2b xenografts (Fig. 4, top panel).

### Endogenous AA metabolism in human prostate cancer xenograft tissues

AA metabolites were further examined in xenografts of the DU145, LNCaP, PC-3, and MDA PCa 2b human prostate cancer cells (Fig. 4, bottom panel). In a pattern similar to that observed in the cultured prostate cancer cell lines, the concentration of endogenous PGE_2_ was significantly higher in PC-3 xenograft tissues (10.5±2.17 ng/mg protein) than it was in the other three xenograft tissues (range, 0.07–1.1 ng/mg of protein) (p<0.001) (Fig. 4A, bottom panel). The levels of 12-HETE in both PC-3 and MDA PCa 2b xenograft tissues were also much higher (13.9 and 10.9 ng/mg protein, respectively) than those in the LNCaP xenograft tissues (3.9 ng/mg protein) (p<0.01) (Fig. 4B, bottom panel). The highest levels of both 5- and 15-HETE in PC3 and MDA PCa 2b tumor tissues were only about one third or one fourth (2.07±0.46 for 5-HETE and 3.24±0.45 ng/mg protein) the levels of PGE_2_ and 12-HETE (Fig. 4C and D, bottom panel). These results appear to correspond with the endogenous levels of these AA metabolites in the same prostate cancer cells *in vitro*.

### Expression of COXs and LOXs in prostate core biopsy specimens containing tumor

**Figure 5:**
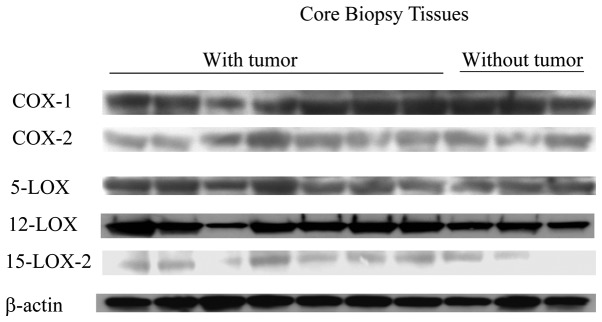
Expression of COX and LOX enzymes in representative *ex vivo* core biopsy samples from prostatectomy specimens. The biopsy specimens were pulverized in a liquid nitrogen-cooled mortar and lysed with buffer as described in Materials and methods. β-actin was used as loading control.

To test the role of AA metabolism in prostate cancer, the expression of COX and LOX proteins in human prostate core biopsy specimens containing tumor was compared with that in normal, nontumorous prostate tissue specimens. As shown in Fig. 5, the expression of COX-1 and COX-2 enzymes did not differ between the tumorous and normal tissues. In almost all of the tumorous specimens, the expression of 12-LOX was moderately greater than it was in the specimens of normal tissue. The protein levels of 5-LOX were also greater in the cores containing tumor than in those without, but the magnitude of the increases was not as remarkable as that in the case of 12-LOX. We found that the expression of 15-LOX-2 was slightly higher in most of the tumorous cores than it was in the normal cores.

### Eicosanoid profile in human prostate core biopsy samples

**Figure 6:**
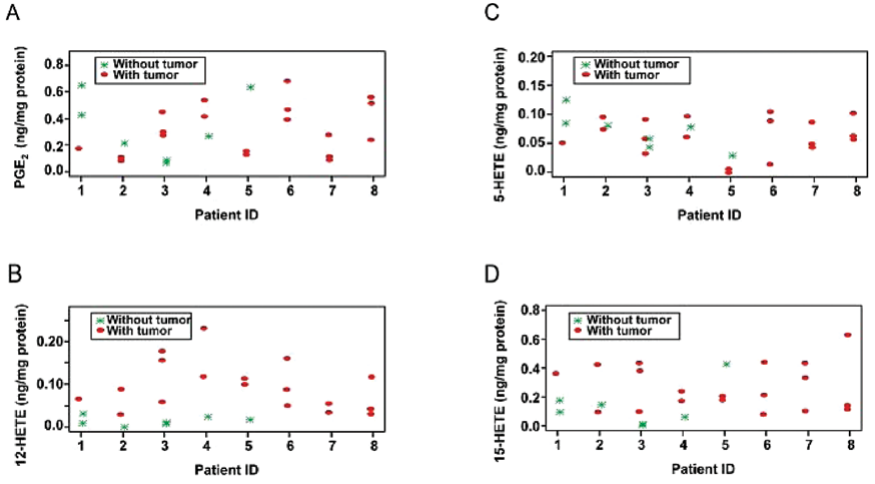
Endogenous eicosanoid profile in *ex vivo* core biopsy samples from prostatectomy specimens. Owing to the limited amount of core biopsy tissues, the specimens were directly lysed with buffer and subjected to the extraction and analysis of arachidonic acid metabolites. The levels of endogenous eicosanoids were normalized by the amount of protein. The data points represent the mean value of duplicate injections of each core biopsy specimen tested. Green asterisks, 7 specimens without tumor; red circles, 19 specimens containing tumor.

AA metabolism was also examined in seven specimens without tumor and 19 specimens containing tumor obtained from core biopsies of prostatectomy specimens from eight patients. A mixed-effects linear model was fitted to assess the differences between the tumorous and normal specimens. The mean PGE_2_ levels in the non-tumor-containing and tumorous specimens were very similar, as shown in Fig. 6A (0.335 ng/mg and 0.314 ng/mg protein, respectively; p=0.703). The mean level of endogenous 12-HETE was significantly higher in the cores containing tumor than it was in those without (0.094 ng/mg and 0.010 ng/mg protein, respectively; p=0.019) (Fig. 6B).

As with the PGE_2_ levels, the mean 5-HETE levels were very similar in tumorous and normal tissues (0.062 ng/mg and 0.073 ng/mg protein, respectively; p= 0.483) (Fig. 6C). However, the mean level of the 15-LOX-2 metabolite 15-HETE in the tumorous cores was more than twice that in the normal cores, although the difference was not statistically significant (0.269 ng/mg and 0.129 ng/mg protein, respectively; p=0.110) (Fig. 6D).

The changes in AA metabolism in the core biopsy specimens containing malignant cells were similar to those observed both in prostate cancer cells *in vitro* and in prostate cancer xenograft tissues, suggesting that 12-LOX may play an important role in the progression of human prostate cancer.

## Discussion

AA-derived COX and LOX metabolites play a critical role in prostate cancer progression. In this study, we found higher levels of 12-HETE in the androgen-independent metastatic prostate cancer cells and their corresponding xenograft tissues than in the androgen-dependent prostate cancer cells. More striking was the finding that among the five AA metabolites examined, the level of endogenous 12-HETE was significantly more elevated in core biopsy specimens containing tumor than it was in those without tumor. Our results also suggest that the COX and LOX enzyme metabolic pathways are modulated differently by exogenous and endogenous AA and that this modulation may be cell type and microenvironment specific.

Previous evidence from specimens obtained during radical prostatectomy showed that AA turnover is 10 times greater in tumor tissue than in normal prostate tissue (7). In addition, the results of multiple studies have supported the idea that AA has a role in the proliferation of prostate cancer cells (8,35,36). Investigators in one recent study reported that AA is critical in modulating the cross talk between prostate carcinoma and bone stromal cells that is mediated by transforming growth factor α, interleukin 1β, and the receptor activator for nuclear factor κB ligand (37), suggesting that AA plays a role in the metastasis of prostate cancer to bone.

Whether COX-2 is involved in prostate cancer progression is controversial. A higher level of expression of COX-2 or its metabolite PGE_2_ has consistently been observed in premalignant lesions of high-grade prostatic intraepithelial neoplasia (PIN) and in benign hyperplastic prostate lesions than is expressed in normal prostate tissues (25,38–40). However, COX-2 expression in cancerous prostate lesions or in more invasive or metastatic disease is more controversial. Larre *et al* (40) reported having found no difference in PGE_2_ production between cancerous prostate samples and adjacent normal tissues. The results of our preclinical evaluations (25) showed that the level of endogenous PGE_2_ was almost five times lower in prostate tissue from TRAMP mice than it was in wild-type mice. Earlier, Shappell *et al* (26) had examined the expression of COX-2 and 15-LOX-2 mRNAs and their related eicosanoid products in human prostate biopsy specimens obtained during radical prostatectomy and found that COX-2 expression was elevated only in high-grade tumors. Our results are in agreement with the previous reported findings with respect to the role of PGE_2_ in prostate cancer progression.

Unlike the case with COX-2, the influence of the LOXs (particularly 5-LOX and 12-LOX) and their metabolites on the progression of prostate cancer has been extensively investigated. We and others have examined alterations of the AA metabolites in the TRAMP mouse model and found that 12-HETE was significantly greater in the prostate tissues of these mice than it was in control or wild-type mice (25,41). The protein and mRNA of 12-LOX are overexpressed in human prostate cancer, and its staining intensity on immunohistochemical testing corresponded with that of advanced-stage or high-grade cancer (42). Furthermore, other investigators have suggested that 12-LOX is related to the metastatic potential of prostate cancer since the expression of 12-LOX was higher in prostate cancer cell lines that metastasized in their corresponding xenograft models (e.g., DU145) than its expression was in PC-3 nm, a nonmetastatic subline of the PC-3 cell line (43). Overexpression of 12-LOX also promotes the angiogenic activity and invasion and metastatic potential of PC-3 cells. When PC-3 cells being transiently transfected with 12-LOX were injected into immunodeficient mice, the cells grew more rapidly and were significantly more angiogenic than nontransfected control PC-3 cells (44). In addition, we previously found that Zyflamend, a multiherbal anti-inflammatory product (New Chapter) had a strong ability to downregulate both 12-LOX and 12-HETE and that this correlated with the product’s ability to inhibit proliferation of PC-3 prostate cancer cells (33). This new study demonstrates for the first time the overexpression of 12-LOX and the higher level of production of its AA metabolite 12-HETE (relative to its production in normal prostate tissues) in highly metastatic prostate cancer cells, at least PC-3 and MDA PCa 2b cells *in vitro*, as well as in corresponding xenografts and tissues from malignant human prostate tumors. These findings thus suggest that 12-LOX and its product 12-HETE have important roles in prostate cancer and moreover that they may be useful as targets in the treatment and prevention of prostate cancer.

It has been suggested (45) that the 5-LOX and 12-LOX pathways are even more potent than the COX-2 pathways and that inhibiting these LOX pathways would prove a more effective therapeutic strategy than inhibiting COX-2 pathways. Our findings agree. Among our five prostate cancer cell lines and their four xenograft models, only PC-3 cells and the related xenograft models showed relatively high levels of COX-2 expression and formation of its metabolite PGE_2_. Furthermore, the COX-2 and PGE_2_ levels in the core biopsy specimens containing tumor were similar to those of the core biopsy specimens without tumor. The expression of mRNA and protein of COX-2 correlated well with the level of PGE_2_ in the prostate cancer cells, the related xenograft tumors, and the human prostate biopsy specimens with tumor. By contrast, the LOX enzyme expression and metabolite levels in the different materials (i.e., cultured cells, xenograft models, and core biopsy specimen cores with tumors) were highly elevated in a number of the prostate cancer cell lines tested. The notable variation in LOX expression and formation of its products under different microenvironmental conditions suggested that modulation of COX and LOX enzyme pathways differs, depending on their microenvironments and whether the source of AA is exogenous or endogenous. This, in turn, suggests that therapeutic interventions targeting these pathways will be more effective than inhibiting a specific enzyme. For example, the mRNA levels of 5-, 12-, and 15-LOXs appear to be positively correlated with the endogenous levels of their relevant metabolites, whereas the exogenous levels of LOX products were proportional to those of the LOX protein (Figs. 1–3). This discord might be due to the sensitivity of antibodies related to each LOX of interest; the cofactors needed for production of eicosanoids, and the relative affinities of each enzyme for their substrate, AA. Thus, these data suggest that evaluating the expression of these eicosanoid enzymes as well as their relevant metabolites in the human biopsy samples is important for delineating the role of these lipid mediators in the development and progression of prostate cancer.

In spite of our efforts and those of other investigators, the involvement of 15-LOX in the initiation and development of prostate cancer remains undefined. It appears that two isoforms of 15-LOX, designated 15-LOX-1 and 15-LOX-2, have different functions in tumor cell growth and survival. For example, the level of 15-LOX-1 is thought to be higher in high-grade PIN lesions than in normal prostate tissue (45), and its overexpression may reflect its activity as a promoter of the development of PIN lesions (46). By contrast, the expression of 15-LOX-2 is noted to be lower in human prostate cancer and high-grade PIN than in normal tissue (47,48). Also, 15-LOX-2 expression was lost in all the prostate cancer cell lines tested by Tang *et al* (17) and Bhatia *et al* (27), including PC-3, LNCaP, and DU145 cells, although it was expressed in all the normal human prostate cells. Other investigators have suggested that the 15-LOX product 15-HETE plays a role in suppressing apoptosis. Our results from this study demonstrate that 15-HETE levels and 15-LOX-2 expression were actually higher in androgen-independent prostate cancer cells, such as MDA PCa 2a and MDA PCa 2b cells, than they were in the androgen-dependent LNCaP cells; furthermore, expression levels were higher in prostate tumor-containing tissues than they were in the tissues without tumor. Thus, it is apparent that the roles of 15-LOX and its metabolites in the progression of human prostate cancer require further investigation.

The results of evaluating endogenous and exogenous AA metabolites using human prostate cancer cell lines, their corresponding xenograft models, and core biopsy specimens obtained during prostatectomy suggest important roles for these metabolites, particularly those generated through the LOX pathways. These metabolites may serve as valuable markers of prostate cancer prognosis and/or response to prostate cancer therapy, and their pathways may be exploitable as potential therapeutic targets. Owing to the limited number of biopsy specimens we tested in this study, however, the important role of these metabolites, especially the 12- and 15-LOXs and their relevant metabolites, in the development of prostate cancer merit further investigation in a large population study.

## Abbreviations:

AA: arachidonic acid
BHT: butylated hydroxytoluene
COX: cyclooxygenase
FBS: fetal bovine serum
HETE: hydroxyeicosatetraenoic acid
13-HODE: 13-hydroxyoctadecadienoic acid
HPLC: high-pressure liquid chromatography
LC/MS/MS: liquid chromatography/tandem mass spectrometry
LT: leukotriene
LOX: lipoxygenase
PCR: polymerase chain reaction
PG: prostaglandin
PIN: prostatic intraepithelial neoplasia
PPARγ: peroxisome proliferator-activated receptor γ

## References

[1] Baade PD, Youlden DR, Krnjacki LJ (2009). International epidemiology of prostate cancer: geographical distribution and secular trends. Mol Nutr Food Res.

[2] Coleman MP, Quaresma M, Berrino F (2008). Cancer survival in five continents: a worldwide population-based study (CONCORD). Lancet Oncol.

[3] Crowe FL, Key TJ, Appleby PN (2008). Dietary fat intake and risk of prostate cancer in the European Prospective Investigation into Cancer and Nutrition. Am J Clin Nutr.

[4] Bidoli E, Talamini R, Bosetti C (2005). Macronutrients, fatty acids, cholesterol and prostate cancer risk. Ann Oncol.

[5] Rose DP, Connolly JM (1991). Effects of fatty acids and eicosanoid synthesis inhibitors on the growth of two human prostate cancer cell lines. Prostate.

[6] Pandalai PK, Pilat MJ, Yamazaki K, Naik H, Pienta KJ (1996). The effects of omega-3 and omega-6 fatty acids on in vitro prostate cancer growth. Anticancer Res.

[7] Chaudry AA, Wahle KW, McClinton S, Moffat LE (1994). Arachidonic acid metabolism in benign and malignant prostatic tissue in vitro: effects of fatty acids and cyclooxygenase inhibitors. Int J Cancer.

[8] Anderson KM, Wygodny JB, Ondrey F, Harris J (1988). Human PC-3 prostate cell line DNA synthesis is suppressed by eicosatetraynoic acid, an in vitro inhibitor of arachidonic acid metabolism. Prostate.

[9] Berquin IM, Min Y, Wu R (2007). Modulation of prostate cancer genetic risk by omega-3 and omega-6 fatty acids. J Clin Invest.

[10] Higgs GA, Moncada S, Vane JR (1984). Eicosanoids in inflammation. Ann Clin Res.

[11] Wallace JM (2002). Nutritional and botanical modulation of the inflammatory cascade--eicosanoids, cyclooxygenases, and lipoxygenases--as an adjunct in cancer therapy. Integr Cancer Ther.

[12] Hughes-Fulford M, Chen Y, Tjandrawinata RR (2001). Fatty acid regulates gene expression and growth of human prostate cancer PC-3 cells. Carcinogenesis.

[13] Subbarayan V, Sabichi AL, Llansa N, Lippman SM, Menter DG (2001). Differential expression of cyclooxygenase-2 and its regulation by tumor necrosis factor-alpha in normal and malignant prostate cells. Cancer Res.

[14] Tang K, Honn KV (1999). 12(S)-HETE in cancer metastasis. Adv Exp Med Biol.

[15] Yin B, Yang Y, Zhao Z (2011). Arachidonate 12-lipoxygenase may serve as a potential marker and therapeutic target for prostate cancer stem cells. Int J Oncol.

[16] Ghosh J, Myers CE (1998). Inhibition of arachidonate 5-lipoxygenase triggers massive apoptosis in human prostate cancer cells. Proc Natl Acad Sci USA.

[17] Tang S, Bhatia B, Maldonado CJ (2002). Evidence that arachidonate 15-lipoxygenase 2 is a negative cell cycle regulator in normal prostate epithelial cells. J Biol Chem.

[18] Shureiqi I, Chen D, Lee JJ (2000). 15-LOX-1: a novel molecular target of nonsteroidal anti-inflammatory drug-induced apoptosis in colorectal cancer cells. J Natl Cancer Inst.

[19] Vanaja DK, Grossmann ME, Celis E, Young CY (2000). Tumor prevention and antitumor immunity with heat shock protein 70 induced by 15-deoxy-delta12,14-prostaglandin J2 in transgenic adenocarcinoma of mouse prostate cells. Cancer Res.

[20] Wang MT, Honn KV, Nie D (2007). Cyclooxygenases, prostanoids, and tumor progression. Cancer Metastasis Rev.

[21] Dassesse T, de Leval X, de Leval L, Pirotte B, Castronovo V, Waltregny D (2006). Activation of the thromboxane A2 pathway in human prostate cancer correlates with tumor Gleason score and pathologic stage. Eur Urol.

[22] Nie D, Guo Y, Yang D (2008). Thromboxane A2 receptors in prostate carcinoma: expression and its role in regulating cell motility via small GTPase Rho. Cancer Res.

[23] Nie D, Che M, Zacharek A (2004). Differential expression of thromboxane synthase in prostate carcinoma: role in tumor cell motility. Am J Pathol.

[24] Wang X, Colby JK, Yang P, Fischer SM, Newman RA, Klein RD (2008). The resistance to the tumor suppressive effects of COX inhibitors and COX-2 gene disruption in TRAMP mice is associated with the loss of COX expression in prostate tissue. Carcinogenesis.

[25] Shappell SB, Olson SJ, Hannah SE (2003). Elevated expression of 12/15-lipoxygenase and cyclooxygenase-2 in a transgenic mouse model of prostate carcinoma. Cancer Res.

[26] Shappell SB, Manning S, Boeglin WE (2001). Alterations in lipoxygenase and cyclooxygenase-2 catalytic activity and mRNA expression in prostate carcinoma. Neoplasia.

[27] Bhatia B, Maldonado CJ, Tang S (2003). Subcellular localization and tumor-suppressive functions of 15-lipoxygenase 2 (15-LOX2) and its splice variants. J Biol Chem.

[28] Navone NM, Olive M, Ozen M (1997). Establishment of two human prostate cancer cell lines derived from a single bone metastasis. Clin Cancer Res.

[29] Pfaffl MW (2001). A new mathematical model for relative quantification in real-time RT-PCR. Nucleic Acids Res.

[30] Shureiqi I, Zuo X, Broaddus R (2007). The transcription factor GATA-6 is overexpressed in vivo and contributes to silencing 15-LOX-1 in vitro in human colon cancer. FASEB J.

[31] Kempen EC, Yang P, Felix E, Madden T, Newman RA (2001). Simultaneous quantification of arachidonic acid metabolites in cultured tumor cells using high-performance liquid chromatography/electrospray ionization tandem mass spectrometry. Anal Biochem.

[32] Yang P, Felix E, Madden T, Fischer SM, Newman RA (2002). Quantitative high-performance liquid chromatography/electrospray ionization tandem mass spectrometric analysis of 2- and 3-series prostaglandins in cultured tumor cells. Anal Biochem.

[33] Yang P, Chan D, Felix E (2006). Determination of endogenous tissue inflammation profiles by LC/MS/MS: COX- and LOX-derived bioactive lipids. Prostaglandins Leukot Essent Fatty Acids.

[34] Yang P, Collin P, Madden T (2003). Inhibition of proliferation of PC3 cells by the branched-chain fatty acid, 12-methyltetradecanoic acid, is associated with inhibition of 5-lipoxygenase. Prostate.

[35] Dahiya R, Yoon WH, Boyle B, Schoenberg S, Yen TS, Narayan P (1992). Biochemical, cytogenetic, and morphological characteristics of human primary and metastatic prostate cancer cell lines. Biochem Int.

[36] Wang Y, Corr JG, Thaler HT, Tao Y, Fair WR, Heston WD (1995). Decreased growth of established human prostate LNCaP tumors in nude mice fed a low-fat diet. J Natl Cancer Inst.

[37] Angelucci A, Garofalo S, Speca S (2008). Arachidonic acid modulates the crosstalk between prostate carcinoma and bone stromal cells. Endocr Relat Cancer.

[38] Zha S, Gage WR, Sauvageot J (2001). Cyclooxygenase-2 is up-regulated in proliferative inflammatory atrophy of the prostate, but not in prostate carcinoma. Cancer Res.

[39] Kirschenbaum A, Klausner AP, Lee R (2000). Expression of cyclooxygenase-1 and cyclooxygenase-2 in the human prostate. Urology.

[40] Larre S, Tran N, Fan C (2008). PGE2 and LTB4 tissue levels in benign and cancerous prostates. Prostaglandins Other Lipid Mediat.

[41] Wang H, Wang X, Zeng A, Yang K (2007). Effects of coherence on anisotropic electromagnetic Gaussian-Schell model beams on propagation. Opt Lett.

[42] Gao LJ, Wang XF, Yang HY, Gao XZ, Lu YC, Cui ZJ (2007). Effects of a lactic acid bacteria community SFC-2 treated on rice straw. Huan Jing Ke Xue.

[43] Timar J, Raso E, Dome B (2000). Expression, subcellular localization and putative function of platelet-type 12-lipoxygenase in human prostate cancer cell lines of different metastatic potential. Int J Cancer.

[44] Nie D, Hillman GG, Geddes T (1998). Platelet-type 12-lipoxygenase in a human prostate carcinoma stimulates angiogenesis and tumor growth. Cancer Res.

[45] Kelavkar UP, Harya NS, Hutzley J (2007). DNA methylation paradigm shift: 15-lipoxygenase-1 upregulation in prostatic intraepithelial neoplasia and prostate cancer by atypical promoter hypermethylation. Prostaglandins Other Lipid Mediat.

[46] Kelavkar UP, Parwani AV, Shappell SB, Martin WD (2006). Conditional expression of human 15-lipoxygenase-1 in mouse prostate induces prostatic intraepithelial neoplasia: the FLiMP mouse model. Neoplasia.

[47] Shappell SB, Boeglin WE, Olson SJ, Kasper S, Brash AR (1999). 15-lipoxygenase-2 (15-LOX-2) is expressed in benign prostatic epithelium and reduced in prostate adenocarcinoma. Am J Pathol.

[48] Gonzalez AL, Roberts RL, Massion PP, Olson SJ, Shyr Y, Shappell SB (2004). 15-Lipoxygenase-2 expression in benign and neoplastic lung: an immunohistochemical study and correlation with tumor grade and proliferation. Hum Pathol.

